# Comparative transcriptome analysis reveals genetic diversity in the endosymbiont *Hamiltonella* between native and exotic populations of *Bemisia tabaci* from Brazil

**DOI:** 10.1371/journal.pone.0201411

**Published:** 2018-07-27

**Authors:** Bruno Rossitto De Marchi, Tonny Kinene, James Mbora Wainaina, Renate Krause-Sakate, Laura Boykin

**Affiliations:** 1 UNESP–Universidade Estadual Paulista, Faculdade de Ciências Agronomicas, Botucatu-SP, Brazil; 2 School of Molecular Sciences and Australian Research Council Centre of Excellence in Plant Energy Biology, University of Western Australia, Crawley, Perth, WA, Australia; Agricultural Research Organization Volcani Center, ISRAEL

## Abstract

The whitefly, *Bemisia tabaci*, is a species complex of more than 40 cryptic species and a major agricultural pest. It causes extensive damage to plants mainly by transmitting plant viruses. There is still a lack of genomic data available for the different whitefly species found in Brazil and their bacterial endosymbionts. Understanding the genetic and transcriptomic composition of these insect pests, the viruses they transmit and the microbiota is crucial to sustainable solutions for farmers to control whiteflies. Illumina RNA-Seq was used to obtain the transcriptome of individual whiteflies from 10 different populations from Brazil including Middle East-Asia Minor 1 (MEAM1), Mediterranean (MED) and New World 2 (NW2). Raw reads were assembled using CLC Genomics Workbench and subsequently mapped to reference genomes. We obtained whitefly complete mitochondrial genomes and draft genomes from the facultative bacterial endosymbiont *Hamiltonella* for further phylogenetic analyses. In addition, nucleotide sequences of the GroEL chaperonin gene from *Hamiltonella* from different populations were obtained and analysed. There was concordance in the species clustering using the whitefly complete mitogenome and the mtCOI gene tree. On the other hand, the phylogenetic analysis using the 12 ORF’s of *Hamiltonella* clustered the native species NW2 apart from the exotics MEAM1 and MED. In addition, the amino acid analysis of GroEL chaperonin revealed a deletion only in *Hamiltonella* infecting NW2 among whiteflies populations analysed which was further confirmed by PCR and Sanger sequencing. The genomic data obtained in this study will aid understanding the functions that *Hamiltonella* may have in whitefly biology and serve as a reference for further studies regarding whiteflies in Brazil.

## Introduction

The whitefly *Bemisia tabaci* (Gennadius) (Hemiptera: Aleyrodidae) is an insect pest of significant economic importance of a wide variety of agricultural crops such as tomatoes, beans, cassava, cotton and ornamentals [1]. *B*. *tabaci* causes damage directly through feeding and indirectly through the transmission of plant pathogenic viruses, belonging to the genera *Begomovirus*, *Carlavirus*, *Crinivirus*, *Ipomovirus* and *Torradovirus* [2]. Currently, *B*. *tabaci* is classified as a cryptic species complex composed of at least 40 morphologically indistinguishable species on the basis of the mtCOI gene [3]. In Brazil, four *B*. *tabaci* species have been reported to date, the natives species from the Americas, New World 1 (NW1) and New World 2 (NW2) and the exotic species Middle East-Asia Minor 1 (MEAM1) and Mediterranean (MED) [4–6]. The exotics, MEAM1 and MED are globally distributed and the most challenging to control [7,8]. Species of the complex *B*. *tabaci* differ in several aspects including the range of host plants utilized, the capacity to cause plant disorders, attraction of natural enemies, response to pesticides and plant virus transmission capabilities [1,9]. Brazil it’s a country with extensive territory and high diversification of the agricultural systems which makes the identification and tracking of pests more complex. The first report of MED in Brazil [4] followed by a second incursion of this species associated to ornamentals plants [10] has highlighted the need of reference data, such as complete mitochondrial genome, to prevent and track the introduction of exotic pests to the country. In addition, full mitogenome comparisons provides a better understanding of evolutionary genetic relationships between members of *B*. *tabaci* [11]. Currently, there are 13 complete whitefly mitochondrial genomes characterized and available on GenBank [11–14].

The complexity of *B*. *tabaci* might also depend on the inherited bacterial endosymbionts whose functions are not fully understood [15]. The obligatory endosymbionts are essential for insects to live on nutritionally poor diets [16,17]. In whiteflies, the obligatory endosymbionts is *Portiera aleyrodidarum* [13]. In addition, insects often harbor facultative endosymbionts that can play different roles on the vector such as enhancing insecticide susceptibility [18,19], facilitating virus transmission [20,21], conferring tolerance to high-temperature [22] and resistance to natural enemies like parasitic wasps [23]. These bacteria have probably been acquired more recently than obligatory endosymbionts [16,24]. In *B*. *tabaci*, different facultative symbionts have been described, including *Arsenophonus*, *Hamiltonella*, *Wolbachia*, *Cardinium*, *Fritschea* and *Rickettsia* [8,20]. Among the facultative symbionts, *Hamiltonella defensa* is a maternally transmitted gamma-proteobacterium found sporadically in sap-feeding insects, including aphids, psyllids, and whiteflies [25–27]. Previous studies in Brazil have shown that different species of *B*. *tabaci*, such as MEAM1, MED and NW2 harbor *Hamiltonella* [10,28]. More recently, a survey has reported that *Hamiltonella* is highly distributed throughout the Brazilian territory and was detected in 89,5% of the MEAM1 specimens and approximately 50% of the MED specimens analyzed [29]. The high incidence of *Hamiltonella* in populations of whiteflies in Brazil may have serious implications to virus transmission. The GroEL proteins encoded by *Hamiltonella* has been found in Israeli *B*. *tabaci* (MEAM1) populations interacting with the coat protein of begomovirus and therefore facilitating virus transmission [20,24]. The GroEL produced by other symbionts of *B*. *tabaci* (MEAM1 and MED) did not interact with the virus and therefore were not involved in virus transmission [20]. *Hamiltonella* can play different roles depending on the vector species. In pea aphids, *Acyrthosiphon pisum* (Harris), *Hamiltonella* can block larval development of the solitary endoparasitoid wasps *Aphidius ervi* and *Aphidius eadyi*, rescuing the aphid host [30].

In this study, we sequenced ten transcriptomes of single whitefly specimens from different Brazilian populations and characterized the full mitochondrial genome belonging to three different species (MEAM1, MED and NW2) of the *B*. *tabaci* complex followed by phylogenetic analysis. In addition, the diversity of the facultative endosymbiont *Hamiltonella* was inferred analyzing 12 different ORF’s. The GroEL amino acid sequences of *Hamiltonella* from different *B*. *tabaci* species were also analyzed. Our goal was to add further details concerning phylogenetic diversity of mitochondrial genome and *Hamiltonella* among Brazilian populations of *B*. *tabaci*. In addition, a GroEL protein analysis was carried out that may give further insights about the functions that *Hamiltonella* may have in whitefly biology.

## Methods

### Whitefly sampling

Samples were obtained from pure colonies and straight from the field. Four different *B*. *tabaci* populations (153, 154, 156 and 320) were analysed, including two exotics species: Middle East-Asia Minor (MEAM1) and Mediterranean (MED); and a native species: New World 2 (NW2). Populations from colonies were previously identified by sequencing and analysis of the mtCOI gene using the primers C1-J-2195 and TL2-N-3014 [31].

### RNA extraction

The RNA extraction of a single individual whitefly was carried out using the ARCTURUS PicoPure kit with modifications [32]. Extracted RNA was subjected to DNase treatment using the TURBO DNA free kit as described by the manufacturer (Ambion Life Technologies CA, USA). Subsequently, the RNA was concentrated using a vacuum centrifuge (Eppendorf, Germany) at 25°C for one hour. The pellet was resuspended in 18 μl of RNase free water and stored at—80 C waiting further analysis. Integrity of RNA was quantified by 2100 Bio-analyser (Aligent Technologies).

### cDNA and Illumina library preparation

Total RNA from each individual whitefly sample was used for cDNA library preparation using the Illumina TruSeq Stranded Total RNA Preparation kit as described by the manufacturer (Illumina, San Diego, CA, USA). Later on, sequencing of 10 samples was carried out using the HiSeq2000 on a rapid run mode generating 2x50 bp paired end reads. Base calling, quality assessment and image analysis were conducted using the HiSeq control software v1.4.8 and Real Time Analysis v1.18.61 at the Macrogen Korea.

### Trimming and *de novo* sequence assembly

The raw transcriptome data was trimmed using the software CLC Genomics Workbench v8.5.1 (CLCGW) with quality scores limit set to 0.01, ambiguous limit set to 2. Trimmed reads were then assembled into contigs using *de novo* sequence assembly tool in CLCGW. The assembly parameters consisted of mismatch cost (2), insertion cost (3), deletion cost (3), length fraction (0.5), similarity fraction (0.9) and minimum contig length of either 500 bp or 1000 bp.

### Obtaining and analysing complete mitochondrial genomes

The whiteflies complete mitochondrial genome was obtained by mapping of the assembled contigs to reference genomes from GenBank using the software Geneious v9.1.3 [33]. For each *B*. *tabaci* species of the complex, a different reference mitogenome was used: KU877168 for MEAM1, JQ906700 for MED and AY521259 for NW2. When the mapped contigs did not covering the full length of the mitochondrial genome of the reference sequence, we resorted to mapping trimmed reads to the reference sequence and thus the whole length of the reference was covered. Mapping was performed with the following setting in Geneious software; minimum overlap 10%, minimum overlap identity 80%, allow gaps 10%, fine tuning set to iterate up to 10 times at custom sensitivity. A consensus between the mapped trimmed reads and the reference was used to form new mitochondrial genome. Improvements on the draft mitochondrial genomes were carried out using the software Pilon, a tool for genome assembly improvement [34]. Subsequently, mitochondrial genomes were annotated by MITOS [35]. Other whiteflies mitogenomes sequences were downloaded from GenBank (KJ778614, KX714967, KY951451, KF734668, KR819174, KY951448, JQ906700, KY951447, KU877168, KY951449. KY951450, KY951452 and AY521259) [11,12,14,15,36–38] and added to the analysis. Sequences obtained were aligned using MAFFT v7.309 [39] followed by visualization and analysis in Geneious software. A total of 19 sequences were aligned and analysed.

### Analysing *Hamiltonella* genetic diversity

Assembled contigs as well as trimmed reads from each sample were mapped to a *Hamiltonella* reference genome (CP016303) to obtain the facultative endosymbiont draft genome. Mapping was performed with the following setting in Geneious software; minimum overlap 10%, minimum overlap identity 80%, allow gaps 10%, fine tuning set to iterate up to 10 times at custom sensitivity. Afterward, draft genomes were aligned to the reference using the whole genome alignment tool LASTZ version 1.02.00 [40] within Geneious v. 9.1.8. Genes with full coverage for all the samples were selected for further Bayesian phylogenetic analysis using the software ExaBayes version 1.4.1 [41].

### Chaperonin GroEL gene analysis

Trimmed reads were mapped onto the reference sequence for chaperonin GroEL gene from *Hamiltonella* of *B*. *tabaci* (AF130421). As the mapped contigs did not cover the full length of the coding region of the reference sequence AF130421, we resorted to mapping trimmed reads to the reference sequence to get the full coverage of the whole length from the reference. A consensus between the mapped trimmed reads and the reference was used to form new chaperonin GroEL sequences, open reading frames (ORF) were predicted in Geneious. In addition, other chaperonin GroEL sequence from *A*. *pisum* was downloaded from Genbank (CP001277) and added to the analysis. Sequences obtained were aligned using MAFFT v7.309 [39] followed by visualization and analysis in Geneious software. A total of 9 sequences were aligned and analysed. In addition, primers were designed (GroEL 1,354 For- CCTC TGCG TCAG ATTG TGGT and GroEL 1,663 Rev–TCAT ACCA TTCA TTCC GCCC A) for a PCR reaction (95°C for 5min, 35 cycles at 95°C for 30 secs, 59.5°C for 30 secs, 72°C for 30 secs and 72°C for 10 min) followed by nucleotide sequencing to confirm the results obtained by NGS.

### Bayesian phylogenetic analyses

All the phylogenetic analyses were run on 384 nodes on the Magnus supercomputer (Pawsey Centre, Western Australia). Mitochondrial genome phylogenetic analysis were performed by MrBayes 3.2.2. [42]. Analyses were run for 30 million generations with sampling every 1000 generations. Each analysis consisted of four independent runs, utilizing four coupled Markov chains. The run convergence was assessed by finding the plateau in the likelihood scores (standard deviation of split frequencies < 0.0015). In each of the runs, the first 25% trees were discarded as burn-in and the posterior probability is shown on each node.

In addition, the *Hamiltonella* phylogenetic analysis was performed on DNA sequences of 12 protein-coding genes for a dataset with 9 taxa using ExaBayes version 1.4.1; [41]. Bayesian analysis was carried out for four independent runs for 1 million generations, with trees sampled every 500 generations. The run convergence was monitored by finding the plateau in the likelihood scores (standard deviation of split frequencies < 0.0015). In each of the runs, the first 25% trees were discarded as burn-in for the estimation of a majority rule consensus topology and posterior probability for each node. Bayesian run files are available from the authors upon request. Trees were visualized, edited and rooted using FigTree v1.4.3.

## Results

The sequenced number of reads from the 10 samples ranged from 29,840,288 to 64,080,188 among the ten samples and the number of contigs assembled ranged from 7,730 to 41,165 (Table 1).

**Table 1:** Next generation sequencing data from single whitefly transcriptomes of *Bemisia tabaci* populations collected in Brazil.

Sample ID	Species	Colony / Open Field	Reference	Number of Reads	Number of Reads After Trimming	CLC minimum contig length	Number of contigs	Contig average length
153_1	MEAM1	Colony	KU877168	36,449,340	36,449,269	Trimmed reads	-	143.9
						500	25,640	1,240
						1000	10,079	2,087
153_2	MEAM1	Colony	N/A	44,093,422	41,041,633	Trimmed reads	-	146.0
						500	31,072	1,228
154_1	MED	Colony	JQ906700	37,206,396	37,206,313	Trimmed reads	-	144.7
						1000	9,737	1,983
154_2	MED	Colony	JQ906700	29,840,288	29,840,226	Trimmed reads	-	144.1
						1000	7,730	1,947
320_1	MED	Open Field	N/A	64,080,188	57,947,512	Trimmed reads	-	145.5
						500	41,165	1,240
320_3	MED	Open Field	JQ906700	39,894,296	39,894,200	Trimmed reads	-	145.1
						1000	9,719	1,969
156_2	NW2	Colony	AY521259	40,826,418	40,826,334	Trimmed reads	-	145.2
						1000	11,961	2,232
156_3	NW2	Colony	AY521259	43,194,264	42,194,175	Trimmed reads	-	145.0
						1000	12,802	2,244
156_4	NW2	Colony	N/A	48,800,318	45,102,719	Trimmed reads	-	145.9
						500	34,090	1,298
156_5	NW2	Colony	N/A	24,568,460	22,491,230	Trimmed reads	-	145.7
						500	21,597	1,310

### *Hamiltonella* genetic diversity

**Fig 1:**
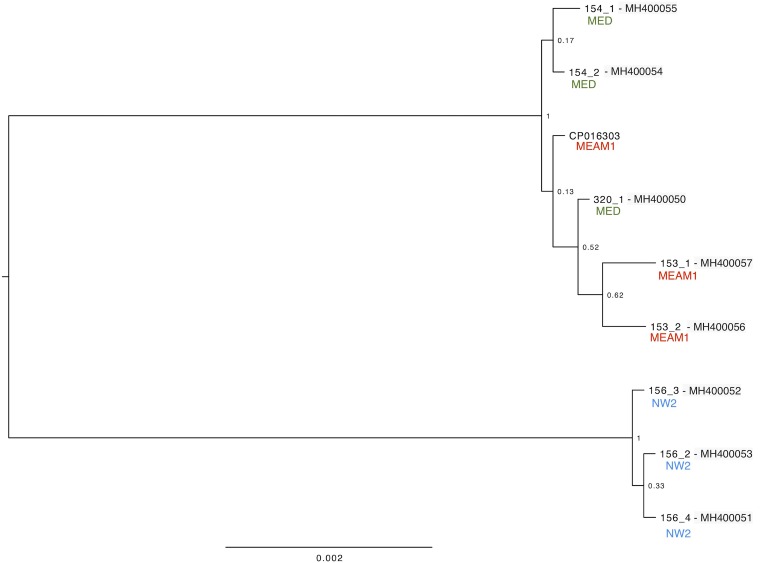
Phylogenetic analysis from 12 ORF’s of *Hamiltonella* from Brazilian populations of *Bemisia tabaci*. Analysis was carried out using the software ExaBayes version 1.4.1. MEAM1, Middle East-Asia Minor-1; MED, Mediterranean; NW2, New World 2.

The diversity of *Hamiltonella* in *B*. *tabaci* populations from Brazil was carried out analysing 12 ORF’s from eight single whitefly transcriptomes totalizing an alignment of 6,378bp length. The analysed ORF’s were: DNA transformation protein tfoX, porin OmpA, acyl carrier, 50S ribosomal protein L3, 50S ribosomal protein L23, 30S ribosomal protein S17, 50S ribosomal protein L24, 50S ribosomal protein L5, rpsN, nucleotide exchange factor GrpE, porin and DNA-binding protein. The phylogenetic analysis from the sequenced accessions separate into two deeply divergent clades representing the native species from the Americas (NW2) and the exotics (MEAM1 and MED) (Fig 1). Furthermore, the identity percentages among the 12 ORF’s from *Hamiltonella* were obtained (S1 Table).

### GroEL chaperonin analysis

**Fig 2:**
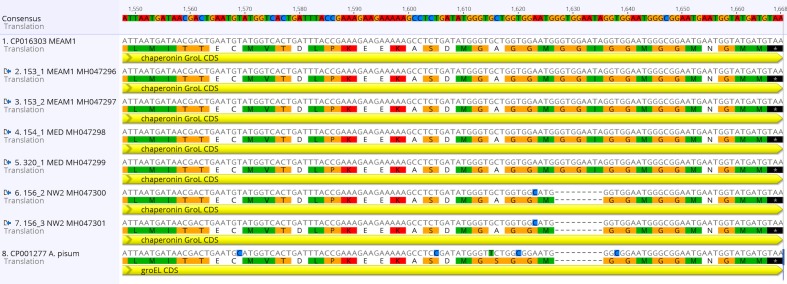
The chaperonin GroEL protein analysis of the *Hamiltonella* endosymbiont. Analysis was visualized on Geneious v9.1.3 and revealed a three amino acids deletion only in the *Bemisia tabaci* New World 2 species and the pea aphid *Acyrthosiphon pisum*.

The GroEL (Chaperonin 60) gene was obtained from six *B*. *tabaci* single whitefly transcriptomes of Brazil. In addition to the data obtained in this study, the analysis also included *Hamiltonella* GroEL sequences downloaded from GenBank from the pea aphid *A*. *pisum* (CP001277) and from *B*. *tabaci* MEAM1 (CP016303). Analysis of the translated proteins revealed a three amino acids deletion present only in the *B*. *tabaci* NW2 species and in the pea aphid *A*. *pisum* (Fig 2). The deletion present in *Hamiltonella* from NW2 and pea aphid was a sequence of two glycine and one isoleucine. The nucleotide deletion was confirmed by PCR, which amplified an 300bp amplicon, followed by nucleotide sequencing.

### Mitochondrial genome

**Fig 3:**
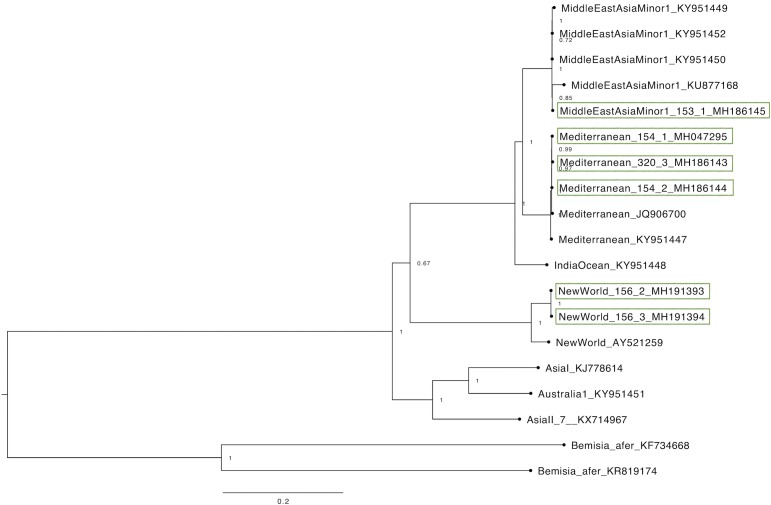
Phylogenetic analysis of the complete mitochondrial genome of whiteflies populations from Brazil obtained from single whitefly transcriptomes. The alignment totalized 19 samples and 16073bp length including references from GenBank. Brazilian samples obtained in this study were highlighted in green.

Six complete mitochondrial genomes were obtained from three different species found in Brazil (NW2, MEAM1 and MED). Phylogenetic analysis of the complete mitochondrial genomes separated different species of the complex in distinct clades (Fig 3). Furthermore, the identity percentages among all the mitogenomes from *B*. *tabaci* were obtained (S2 Table).

## Discussion

In this study we present the phylogenetic relationship of *B*. *tabaci* and its association with the facultative endosymbiont *Hamiltonella*. We show the evolutionary differences between native populations of *B*. *tabaci* species of the Americas and invasive *B*. *tabaci* species. From our data the genetic differences are not confined to the mtCOI gene, but extend to the rest of the mitogenome and to the facultative endosymbiont *Hamiltonella*. These findings will contribute to the understanding about the functions that *Hamiltonella* may have in *B*. *tabaci* biology and will aid the correct identification of *B*. *tabaci* specimens.

Six complete mitochondrial genomes were obtained from three different species of the complex *B*. *tabaci*: MEAM1, MED and NW2. Complete mitochondrial genomes comparisons are essential for better understanding the evolutionary genetic relationships between members of the *B*. *tabaci* species complex [3,11]. There are 13 complete whitefly mitochondrial genomes characterized and available on GenBank to date [11,12,14,15,36–38] which is a valuable contribution to a better understanding of the taxonomy of this global pest. The addition of six more full mitogenomes from *B*. *tabaci* to the global database will contribute for more accurate identification and will serve as references for further full mitochondrial genome phylogenies studies. The phylogenetic analysis of the full mitogenome (Fig 3) conducted in this study separated the species in different clades in a similar pattern compared to phylogenetic trees of partial fragment of the mtCOI gene [1]. This reinforces that using a partial fragment of the mtCOI gene to infer phylogeny in *B*. *tabaci* is a reliable way to delimitate the species boundaries and it represents the whole mitochondrial genome.

A multilocus phylogenetic analysis was carried out for the facultative endosymbiont *Hamiltonella*. Previous studies in Brazil phylogenetically analyzed *Hamiltonella* based on 16S rRNA gene and found genetically homogeneous populations of *Hamiltonella* from MEAM1 and MED across Brazil [29]. Another phylogenetic study based on 16S rRNA gene, in Southeast Europe, have grouped *Hamiltonella* from both *B*. *tabaci* and *T*. *vaporariorum* in the same clades. Our phylogenetic analysis based on 12 ORF’s clustered *Hamiltonella* from NW2 populations in a different clade from MEAM1 and MED populations (Fig 1) suggesting that native populations are infected with a genetically different *Hamiltonella* from invasive populations, reinforcing the inexistence of endosymbiont horizontal transmission between invasive and native species.

The differences of *Hamiltonella* from native and exotic species extends to the GroEL gene. Our analysis revealed an amino acid deletion of two glycine and one isoleucine in the GroEL gene present only in *Hamiltonella* from native populations (Fig 2). Glycine is a non-essential amino acid and isoleucine is an essential amino acid for *Hamiltonella* [43]. The absence of these amino acids could be affecting the confirmation of GroEL on populations of *Hamiltonella* presenting this deletion. Thus, this deletion in *Hamiltonella* could imply in biological changes in populations of whiteflies harbouring this facultative endosymbiont.

It’s known that facultative bacterial endosymbionts are associated with viral transmission [20,21] and different members of the *B*. *tabaci* species complex may transmit the same viruses with different efficiencies [44]. The facultative endosymbiont *Hamiltonella* has been identified as a key driver in the transmission of begomoviruses [24]. *Hamiltonella* encodes a GroEL chaperonin homologue protein that is crucial in safeguarding begomoviruses in the haemolymph [24]. Several studies have shown the interaction between the begomoviruses *Tomato yellow leaf curl virus* (TYLCV) coat protein (CP) and GroEL present in the haemolymph of *B*. *tabaci* [20,45]. Disturbing the association GroEL-TYLCV in vivo by feeding insects with an antibody raised against *Buchnera* GroEL leads to the degradation of the virus and to a markedly decrease in transmission efficiency of the virus [24,45,46].

There is still a lack of knowledge regarding the interactions among vector, symbionts and whitefly-transmitted viruses within Brazilian populations. Previous surveys conducted in Brazil have found that field populations of MEAM1, MED and NW2 analyzed frequently harbor *Hamiltonella* [28, 41]. Therefore, it would be important to find out if there is a relationship between the diversity of *H*. *defensa* found in the current study and biological features of field populations of *B*. *tabaci*. The existence of biological and behavioral differences between the native species New World and the exotic MEAM1 have been reported already [5,47]. The New World *B*. *tabaci* are found more often colonizing weeds and wild plants [5]. In addition, previous transmission studies comparing NW2 and MEAM1 species, both harboring *Hamiltonella*, have found a different transmission efficiency of Brazilian whitefly-transmitted viruses between the species [47]. It was found that the begomovirus *Euphorbia yellow mosaic virus* (EuYMV) is transmitted more efficiently by NW2 compared to MEAM1 and that the crinivirus, *Tomato chlorosis virus* (ToCV) and the carlavirus, *Cowpea mild mottle virus* (CpMMV) are transmitted more efficiently by MEAM1 than NW2 [47]. Interestingly, each of these viruses has a different mode of transmission by the whitefly vector. The begomoviruses, EuYMV is transmitted in a persistent manner, the crinivirus, ToCV is transmitted in a semipersistent manner and the carlavirus, CpMMV is transmitted in a non-persistent mode [48,49], which is a very unusual mode of transmission among *B*. *tabaci* transmitted viruses.

The reasons for a difference in transmission efficiencies by NW2 and MEAM1 are still unknown but might be related to several factors related to the host, the viruses or other facultative endosymbiont found in the vector. However, the data found in this study of phylogenetic differences between insect symbiotic *Hamiltonella* from NW2 and MEAM1 added to the deletion of three amino acids present in the homologue GroEL Chaperonin protein might aid to explain the difference in the transmission efficiencies among native and exotic species present in Brazil if further studies were carried out.

The genomic data obtained in this study from the facultative endosymbionts, *Hamiltonella* and the complete mitochondrial of Brazilian *B*. *tabaci* populations is unprecedented and essential to serve as a reference for further studies regarding whiteflies in Brazil. The phylogenetic and amino acid analysis revealed genetic diversity between *Hamiltonella* from native and exotic populations that will aid for better understanding about the functions that *Hamiltonella* may have in whitefly biology.

## Supporting information

S1 Table Identity percentage among 12 ORF’s from *Hamiltonella* from different *Bemisia tabaci* species. The identity percentage was obtained on Geneious software v9.1.8.
(DOCX)

S2 Table Identity percentage among *Bemisia tabaci* mitochondrial genomes. The identity percentage was obtained on Geneious v9.1.8.
(DOCX)

## References

[1] De Barro PJ, Liu S-S, Boykin LM, Dinsdale AB. *Bemisia tabaci*: A Statement of Species Status. Annu Rev Entomol. Annual Reviews; 2011;56: 1–19. 10.1146/annurev-ento-112408-085504 20690829

[2] Navas-Castillo J, Fiallo-Olivé E, Sánchez-Campos S. Emerging Virus Diseases Transmitted by Whiteflies. Annu Rev Phytopathol. Annual Reviews; 2011;49: 219–248. 10.1146/annurev-phyto-072910-095235 21568700

[3] Boykin Boykin, Armstrong Karen, Kubatko Laura, De Barro Paul. Species Delimitation and Global Biosecurity. Evol Bioinforma. 2011; 1 10.4137/EBO.S8532 PMC3256992 22267902

[4] da Fonseca Barbosa L, Yuki VA, Marubayashi JM, De Marchi BR, Perini FL, Pavan MA, et al First report of Bemisia tabaci Mediterranean (Q biotype) species in Brazil. Pest Manag Sci. 2015;71 10.1002/ps.3909 25212515

[5] Barbosa LFF, Marubayashi JM, De Marchi BR, Yuki VA, Pavan MAMA, Moriones E, et al Indigenous American species of the Bemisia tabaci complex are still widespread in the Americas. Pest Manag Sci. 2014;70: 1440–1445. 10.1002/ps.3731 24458534

[6] Marubayashi JM, Yuki VA, Rocha KCG, Mituti T, Pelegrinotti FM, Ferreira FZ, et al At least two indigenous species of the *Bemisia tabaci* complex are present in Brazil. J Appl Entomol. Blackwell Publishing Ltd; 2013;137: 113–121. 10.1111/j.1439-0418.2012.01714.x

[7] Gauthier N, Clouet C, Perrakis A, Kapantaidaki D, Peterschmitt M, Tsagkarakou A. Genetic structure of *Bemisia tabaci* Med populations from home-range countries, inferred by nuclear and cytoplasmic markers: impact on the distribution of the insecticide resistance genes. Pest Manag Sci. 2014;70: 1477–1491. 10.1002/ps.3733 24458589

[8] Hu J, De Barro P, Zhao H, Wang J, Nardi F, Liu S-S. An Extensive Field Survey Combined with a Phylogenetic Analysis Reveals Rapid and Widespread Invasion of Two Alien Whiteflies in China. Roberts RG, editor. PLoS One. Public Library of Science; 2011;6: e16061 10.1371/journal.pone.0016061 21283707 PMC3025023

[9] Xu J, De Barro PJ, Liu SS. Reproductive incompatibility among genetic groups of Bemisia tabaci supports the proposition that the whitefly is a cryptic species complex. Bull Entomol Res. 2010;100: 359–366. 10.1017/S0007485310000015 20178675

[10] Aparecida de Moraes L, Marubayashi Massaharu J, Yuki Atsushi V, Ghanim M, Bello VHVH, Rossitto De Marchi B, et al New invasion of Bemisia tabaci Mediterranean species in Brazil associated to ornamental plants. Phytoparasitica. Springer Netherlands; 2017;45: 1–9. 10.1007/s12600-017-0607-9

[11] Tay WT, Elfekih S, Court L, Gordon KH, De Barro PJ. Complete mitochondrial DNA genome of Bemisia tabaci cryptic pest species complex Asia I (Hemiptera: Aleyrodidae). Mitochondrial DNA. 2014;1736: 1–2. 10.3109/19401736.2014.926511 24960562

[12] Wang H-L, Yang J, Boykin LM, Zhao Q-Y, Li Q, Wang X-W, et al The characteristics and expression profiles of the mitochondrial genome for the Mediterranean species of the Bemisia tabaci complex. BMC Genomics. 2013;14: 401 10.1186/1471-2164-14-401 23768425 PMC3691742

[13] Thao ML, Baumann P. Evolutionary relationships of primary prokaryotic endosymbionts of whiteflies and their hosts. Appl Environ Microbiol. American Society for Microbiology; 2004;70: 3401–6. 10.1128/AEM.70.6.3401-3406.2004 15184137 PMC427722

[14] Tay WT, Elfekih S, Polaszek A, Court LN, Evans GA, Gordon KHJ, et al Novel molecular approach to define pest species status and tritrophic interactions from historical Bemisia specimens. Sci Rep. 2017;7: 429 10.1038/s41598-017-00528-7 28348369 PMC5428565

[15] Parrella G, Nappo AG, Manco E, Greco B, Giorgini M. Invasion of the Q2 mitochondrial variant of Mediterranean *Bemisia tabaci* in southern Italy: possible role of bacterial endosymbionts. Pest Manag Sci. 2014;70: 1514–1523. 10.1002/ps.3686 24272923

[16] Baumann P. Biology of Bacteriocyte-Associated Endosymbionts of Plant Sap-Sucking Insects. Annu Rev Microbiol. 2005;59: 155–189. 10.1146/annurev.micro.59.030804.121041 16153167

[17] Ferrari J, Vavre F. Bacterial symbionts in insects or the story of communities affecting communities. Philos Trans R Soc Lond B Biol Sci. The Royal Society; 2011;366: 1389–400. 10.1098/rstb.2010.0226 21444313 PMC3081568

[18] Kontsedalov S, Zchori‐Fein E, Chiel E, Gottlieb Y, Inbar M, Ghanim M. The presence of *Rickettsia* is associated with increased susceptibility of *Bemisia tabaci* (Homoptera: Aleyrodidae) to insecticides. Pest Manag Sci. 2008;64: 789–792. 10.1002/ps.1595 18432613

[19] Kontsedalov S, Gottlieb Y, Ishaaya I, Nauen R, Horowitz R, Ghanim M. Toxicity of spiromesifen to the developmental stages of *Bemisia tabaci* biotype B. Pest Manag Sci. 2009;65: 5–13. 10.1002/ps.1636 18785225

[20] Gottlieb Y, Zchori-Fein E, Mozes-Daube N, Kontsedalov S, Skaljac M, Brumin M, et al The transmission efficiency of tomato yellow leaf curl virus by the whitefly Bemisia tabaci is correlated with the presence of a specific symbiotic bacterium species. J Virol. American Society for Microbiology; 2010;84: 9310–7. 10.1128/JVI.00423-10 20631135 PMC2937599

[21] Rana VS, Singh ST, Priya NG, Kumar J, Rajagopal R. Arsenophonus GroEL Interacts with CLCuV and Is Localized in Midgut and Salivary Gland of Whitefly B. tabaci. Oliveira PL, editor. PLoS One. Public Library of Science; 2012;7: e42168 10.1371/journal.pone.0042168 22900008 PMC3416813

[22] Brumin M, Kontsedalov S, Ghanim M. Rickettsia influences thermotolerance in the whitefly Bemisia tabaci B biotype. Insect Sci. Blackwell Publishing Asia; 2011;18: 57–66. 10.1111/j.1744-7917.2010.01396.x

[23] Mahadav A, Gerling D, Gottlieb Y, Czosnek H, Ghanim M. Parasitization by the wasp Eretmocerus mundus induces transcription of genes related to immune response and symbiotic bacteria proliferation in the whitefly Bemisia tabaci. BMC Genomics. 2008;9: 342 10.1186/1471-2164-9-342 18638407 PMC2488360

[24] Gorovits R, Czosnek H. Insect Symbiotic Bacterial GroEL (Chaperonin 60) and Plant Virus Transmission. In: Mande SC, Kumar CMS, Sharma A, editors. Moonlighting Cell Stress Proteins in Microbial Infections. 2013 pp. 173–187. 10.1007/978-94-007-6787-4

[25] Rao Q, Wang S, Su YL, Bing XL, Liu SS, Wang XW. Draft genome sequence of “Candidatus Hamiltonella defensa,” an endosymbiont of the whitefly Bemisia tabaci [Internet]. Journal of Bacteriology. 2012 p. 3558 10.1128/JB.00069-12 22689243 PMC3434728

[26] Luan J-B, Shan H-W, Isermann P, Huang J-H, Lammerding J, Liu S-S, et al Cellular and molecular remodelling of a host cell for vertical transmission of bacterial symbionts. Proc R Soc B Biol Sci. 2016;283: 20160580 10.1098/rspb.2016.0580 27358364 PMC4936034

[27] Luan J, Sun X, Fei Z, Douglas AE. Maternal Inheritance of a Single Somatic Animal Cell Displayed by the Bacteriocyte in the Whitefly Bemisia tabaci. Curr Biol. 2018;28: 459–465.e3. 10.1016/j.cub.2017.12.041 29395925 PMC5807091

[28] Marubayashi JM, Kliot A, Yuki VA, Rezende JAM, Krause-Sakate R, Pavan MA, et al Diversity and Localization of Bacterial Endosymbionts from Whitefly Species Collected in Brazil. Bourtzis K, editor. PLoS One. Public Library of Science; 2014;9: e108363 10.1371/journal.pone.0108363 25259930 PMC4178154

[29] De Moraes Aparecida L, Muller C, Oliveira de Freitas Bueno RC, Santos A, Bello VH, Rossitto De Marchi B, et al Distribution and phylogenetics of whiteflies in Brazil and their endosymbiont relationships. unpublised. 10.1038/s41598-018-32913-1 PMC6167372 30275487

[30] Su Q, Oliver KM, Pan H, Jiao X, Liu B, Xie W, et al Facultative Symbiont &lt;I&gt;Hamiltonella&lt;/I&gt; Confers Benefits to &lt;I&gt;Bemisia tabaci&lt;/I&gt; (Hemiptera: Aleyrodidae), an Invasive Agricultural Pest Worldwide. Environ Entomol. 2013;42: 1265–1271. 10.1603/EN13182 24280594

[31] Frohlich DR, Torres-Jerez I, Bedford ID, Markham PG, Brown JK. A phylogeographical analysis of the Bemisia tabaci species complex based on mitochondrial DNA markers. Mol Ecol. 1999;8: 1683–1691. 10.1046/j.1365-294x.1999.00754.x 10583831

[32] Sseruwagi P, Wainaina J, Ndunguru J, Tumuhimbise R, Tairo F, Guo J-Y, et al The first transcriptomes from field-collected individual whiteflies (Bemisia tabaci, Hemiptera: Aleyrodidae). Gates Open Res. 2017;1: 16 10.12688/gatesopenres.12783.1 PMC5872585 29608200

[33] Kearse M, Moir R, Wilson A, Stones-Havas S, Cheung M, Sturrock S, et al Geneious Basic: An integrated and extendable desktop software platform for the organization and analysis of sequence data. Bioinformatics. 2012;28: 1647–1649. 10.1093/bioinformatics/bts199 22543367 PMC3371832

[34] Walker BJ, Abeel T, Shea T, Priest M, Abouelliel A, Sakthikumar S, et al Pilon: An Integrated Tool for Comprehensive Microbial Variant Detection and Genome Assembly Improvement. Wang J, editor. PLoS One. Public Library of Science; 2014;9: e112963 10.1371/journal.pone.0112963 25409509 PMC4237348

[35] Bernt M, Donath A, Jühling F, Externbrink F, Florentz C, Fritzsch G, et al MITOS: Improved de novo metazoan mitochondrial genome annotation. Mol Phylogenet Evol. 2013;69: 313–319. 10.1016/j.ympev.2012.08.023 22982435

[36] Tay WT, Elfekih S, Court LN, Gordon KHJ, Delatte H, De Barro PJ. The Trouble with MEAM2: Implications of Pseudogenes on Species Delimitation in the Globally Invasive Bemisia tabaci (Hemiptera: Aleyrodidae) Cryptic Species Complex. Genome Biol Evol. Oxford University Press; 2017;9: 2732–2738. 10.1093/gbe/evx173 28985301 PMC5647793

[37] Wang H-L, Zhang Z, Bing X-L, Liu Y-Q, Liu S-S, Wang X-W. A complete mitochondrial DNA genome derived from a Chinese population of the *Bemisia afer* species complex (Hemiptera: Aleyrodidae). Mitochondrial DNA Part A. 2016;27: 3500–3501. 10.3109/19401736.2015.1066367 26218308

[38] Wang H-L, Xiao N, Yang J, Wang X-W, Colvin J, Liu S-S. The complete mitochondrial genome of *Bemisia afer* (Hemiptera: Aleyrodidae). Mitochondrial DNA. 2016;27: 98–99. 10.3109/19401736.2013.873921 24438292

[39] Katoh K, Misawa K, Kuma K, Miyata T. MAFFT: a novel method for rapid multiple sequence alignment based on fast Fourier transform. Nucleic Acids Res. Oxford University Press; 2002;30: 3059–66. Available: http://www.ncbi.nlm.nih.gov/pubmed/12136088 12136088 10.1093/nar/gkf436 PMC135756

[40] Schwartz S, Kent WJ, Smit A, Zhang Z, Baertsch R, Hardison RC, et al Human–Mouse Alignments with BLASTZ. Genome Res. 2003;13: 103–107. 10.1101/gr.809403 12529312 PMC430961

[41] Aberer AJ, Kobert K, Stamatakis A. ExaBayes: Massively Parallel Bayesian Tree Inference for the Whole-Genome Era. Mol Biol Evol. Oxford University Press; 2014;31: 2553–2556. 10.1093/molbev/msu236 25135941 PMC4166930

[42] Ronquist F, Teslenko M, van der Mark P, Ayres DL, Darling A, H?hna S, et al MrBayes 3.2: Efficient Bayesian Phylogenetic Inference and Model Choice Across a Large Model Space. Syst Biol. 2012;61: 539–542. 10.1093/sysbio/sys029 22357727 PMC3329765

[43] Degnan PH, Yu Y, Sisneros N, Wing RA, Moran NA. Hamiltonella defensa, genome evolution of protective bacterial endosymbiont from pathogenic ancestors. Proc Natl Acad Sci. 2009;106: 9063–9068. 10.1073/pnas.0900194106 19451630 PMC2690004

[44] Polston JE, De Barro P, Boykin LM. Transmission specificities of plant viruses with the newly identified species of the *Bemisia tabaci* species complex. Pest Manag Sci. John Wiley & Sons, Ltd; 2014;70: 1547–1552. 10.1002/ps.3738 24464790

[45] Morin S, Ghanim M, Sobol I, Czosnek H. The GroEL Protein of the Whitefly Bemisia tabaci Interacts with the Coat Protein of Transmissible and Nontransmissible Begomoviruses in the Yeast Two-Hybrid System. Virology. 2000;276: 404–416. 10.1006/viro.2000.0549 11040131

[46] Morin S, Ghanim M, Zeidan M, Czosnek H, Verbeek M, van den Heuvel JFJM. A GroEL Homologue from Endosymbiotic Bacteria of the WhiteflyBemisia tabaciIs Implicated in the Circulative Transmission of Tomato Yellow Leaf Curl Virus. Virology. 1999;256: 75–84. 10.1006/viro.1999.9631 10087228

[47] De Marchi BR, Marubayashi JM, Favara GM, Yuki VA, Watanabe LFLFM, Barbosa LFLF, et al Comparative transmission of five viruses by Bemisia tabaci NW2 and MEAM1. Trop Plant Pathol. Tropical Plant Pathology; 2017;1 10.1007/s40858-017-0186-9

[48] Marubayashi JM, Yuki VA, Wutke EB. Transmission of the Cowpea mild mottle virus by whitefly Bemisia tabaci biotype B for plants of beans and soy. Summa Phytopathol. Grupo Paulista de Fitopatologia; 2010;36: 158–160. 10.1590/S0100-54052010000200009

[49] Zanardo LG, Carvalho CM. Cowpea mild mottle virus (Carlavirus, Betaflexiviridae): a review. Trop Plant Pathol. Tropical Plant Pathology; 2017; 417–430. 10.1007/s40858-017-0168-y

