# Supplementary material for: Comparative transcriptome analysis reveals genetic diversity in the endosymbiont Hamiltonella between native and exotic populations of Bemisia tabaci from Brazil
Source: PLoS One. 2018 Jul 27;13(7):e0201411. doi: 10.1371/journal.pone.0201411 (PMC6063447; doi:10.1371/journal.pone.0201411)
Supplement: S1 Table — The identity percentage was obtained on Geneious software v9.1.8. (DOCX) [file pone.0201411.s001.docx]

|  | MEAM1  153_1  MH400057 | MEAM1  153_2  MH400056 | MED  154_1 MH400055 | MED  154_2 MH400054 | NW2  156_2  MH400053 | NW2  156_3  MH400052 | NW2  156_4  MH400051 | MED  320_1  MH400050 | MEAM1  CP016303 |
| --- | --- | --- | --- | --- | --- | --- | --- | --- | --- |
| MEAM1  153_1  MH400057 | | 99.9 | 100 | 100 | 98.9 | 98.9 | 98.9 | 100 | 100 |
| MEAM1  153_2  MH400056 | 99.9 |  | 99.9 | 99.9 | 98.8 | 98.8 | 98.8 | 99.9 | 99.9 |
| MED  154_1  MH400055 | 100 | 99.9 |  | 100 | 98.9 | 98.9 | 98.9 | 100 | 100 |
| MED  154_2  MH400054 | 100 | 99.9 | 100 |  | 98.9 | 98.9 | 98.9 | 100 | 100 |
| NW2  156_2  MH400053 | 98.9 | 98.8 | 98.9 | 98.9 |  | 100 | 100 | 98.9 | 98.9 |
| NW2  156_3  MH400052 | 98.9 | 98.8 | 98.9 | 98.9 | 100 |  | 100 | 98.9 | 98.9 |
| NW2  156_4  MH400051 | 98.9 | 98.8 | 98.9 | 98.9 | 100 | 100 |  | 98.9 | 98.9 |
| MED  320_1  MH400050 | 100 | 99.9 | 100 | 100 | 98.9 | 98.9 | 98.9 |  | 100 |
| MEAM1  CP016303 | 100 | 99.9 | 100 | 100 | 98.9 | 98.9 | 98.9 | 100 |  |

**S1 Table. Identity percentage among 12 ORF’s from *Hamiltonella* from different *Bemisia tabaci* species.** The identity percentage was obtained on Geneious software v9.1.8.
