# Supplementary material for: Comparative transcriptome analysis reveals genetic diversity in the endosymbiont Hamiltonella between native and exotic populations of Bemisia tabaci from Brazil
Source: PLoS One. 2018 Jul 27;13(7):e0201411. doi: 10.1371/journal.pone.0201411 (PMC6063447; doi:10.1371/journal.pone.0201411)
Supplement: S2 Table — The identity percentage was obtained on Geneious v9.1.8. (DOCX) [file pone.0201411.s002.docx]

|  | MED  154_1  MH047295 | MED  154_2  MH186144 | MED  320_3  MH186143 | MED  JQ906700 | MED  KY951447 | MEAM1  153_1  MH186145 | MEAM1  KU877168 | MEAM1  KY951449 | MEAM1  KY951450 | MEAM1  KY951452 | NW  156_2  MH191393 | NW  156_3  MH191394 | NW  AY521259 |
| --- | --- | --- | --- | --- | --- | --- | --- | --- | --- | --- | --- | --- | --- |
| MED  154_1  MH047295 | | 99.9 | 99.8 | 99.8 | 99.6 | 90.5 | 89.2 | 90.7 | 92.4 | 92.4 | 78.2 | 78.2 | 78.3 |
| MED  154_2  MH186144 | 99.9 |  | 99.8 | 99.8 | 99.6 | 90.5 | 89.2 | 90.7 | 92.4 | 92.4 | 78.2 | 78.1 | 78.3 |
| MED  320_3  MH186143 | 99.8 | 99.8 |  | 99.7 | 99.5 | 90.5 | 89.1 | 90.7 | 92.3 | 92.3 | 78.2 | 78.2 | 78.3 |
| MED  JQ906700 | 99.8 | 99.8 | 99.7 |  | 99.6 | 90.4 | 89.1 | 90.7 | 92.3 | 92.3 | 78.2 | 78.1 | 78.3 |
| MED  KY951447 | 99.6 | 99.6 | 99.5 | 99.6 |  | 90.5 | 89.2 | 90.7 | 92.4 | 92.4 | 78.2 | 78.1 | 78.3 |
| MEAM1  153_1  MH186145 | 90.5 | 90.5 | 90.5 | 90.4 | 90.5 |  | 97.7 | 98.9 | 98 | 98 | 78.3 | 78.2 | 78.5 |
| MEAM1  KU877168 | 89.2 | 89.2 | 89.1 | 89.1 | 89.2 | 97.7 |  | 96.8 | 96.1 | 96 | 78.6 | 78.5 | 79 |
| MEAM1  KY951449 | 90.7 | 90.7 | 90.7 | 90.7 | 90.7 | 98.9 | 96.8 |  | 98.3 | 98.3 | 77.7 | 77.6 | 77.9 |
| MEAM1  KY951450 | 92.4 | 92.4 | 92.3 | 92.3 | 92.4 | 98 | 96.1 | 98.3 |  | 100 | 78 | 77.9 | 78.2 |
| MEAM1  KY951452 | 92.4 | 92.4 | 92.3 | 92.3 | 92.4 | 98 | 96 | 98.3 | 100 |  | 78 | 78 | 78.2 |
| NW  156_2  MH191393 | 78.2 | 78.2 | 78.2 | 78.2 | 78.2 | 78.3 | 78.6 | 77.7 | 78 | 78 |  | 99.9 | 94.8 |
| NW  156_3  MH191394 | 78.2 | 78.1 | 78.2 | 78.1 | 78.1 | 78.2 | 78.5 | 77.6 | 77.9 | 78 | 99.9 |  | 94.7 |
| NW  AY521259 | 78.3 | 78.3 | 78.3 | 78.3 | 78.3 | 78.5 | 79 | 77.9 | 78.2 | 78.2 | 94.8 | 94.7 |  |

**S2 Table. Identity percentage among *Bemisia tabaci* mitochondrial genomes.** The identity percentage was obtained on Geneious v9.1.8.
